# Pulmonary Embolism in Acquired Hemophilia A: A Rare Complication With Factor VIII Inhibitor Bypassing Activity Therapy

**DOI:** 10.7759/cureus.9152

**Published:** 2020-07-12

**Authors:** Hafiz M Aslam, Timothy Chong, Joseph Park, Ted Nicolosi, Rehan Shah

**Affiliations:** 1 Internal Medicine, Hackensack Meridian School of Medicine at Seton Hall University, Nutley, USA; 2 Internal Medicine, Drexel College of Medicine, Philadelphia, USA; 3 Radiology, University at Albany, Albany, USA; 4 Internal Medicine - Rheumatology, St. Francis Medical Center, Trenton, USA

**Keywords:** hemophilia, feiba, pulmonary embolism

## Abstract

Acquired hemophilia A (AHA) is an uncommon complication caused by autoantibodies against Factor VIII. The main concern with these patients is hemorrhage, which is often treated with Factor VIII inhibitor bypassing activity (FEIBA). On rare occasions, treatment with FEIBA can result in thromboembolism, a potentially fatal complication. This unfortunate situation occurred in our patient, a 64-year-old female who was treated with FEIBA after being diagnosed with AHA. After initiating FEIBA, she developed clinical signs and symptoms of pulmonary embolism, which was ultimately responsible for her acute death. While pulmonary embolism may be a rare complication of FEIBA treatment, clinicians should be aware of its possibility, especially as the complete safety profile for this treatment is not well known.

## Introduction

Hemophilia A is an inherited bleeding disorder in which there are reduced or absent levels of clotting Factor VIII, putting affected people at increased bleeding risk due to inadequate blood coagulation. Acquired hemophilia A (AHA) is caused by autoantibodies against the coagulation factor rather than a genetic deficiency. In a survey of 215 patients with AHA, 87% experienced major bleeding, and 22% died from bleeding complications attributed to the autoantibody [1]. In patients with severe bleeding, Factor VIII inhibitor bypassing activity (FEIBA) may be used to promote hemostasis. A rare complication of this therapy is thromboembolism, which led to a clinical diagnosis of pulmonary embolism in our patient.

## Case presentation

Our patient was a 64-year-old female with a history of pulmonary sarcoidosis and Addison’s disease. She presented to the ED with a two-day history of progressively worsening painful swelling in her left upper thigh. She had been experiencing bruising in various parts of her body for the last two years prior to presentation. She denied any familial or personal history of bleeding disorders. 

**Figure 1:**
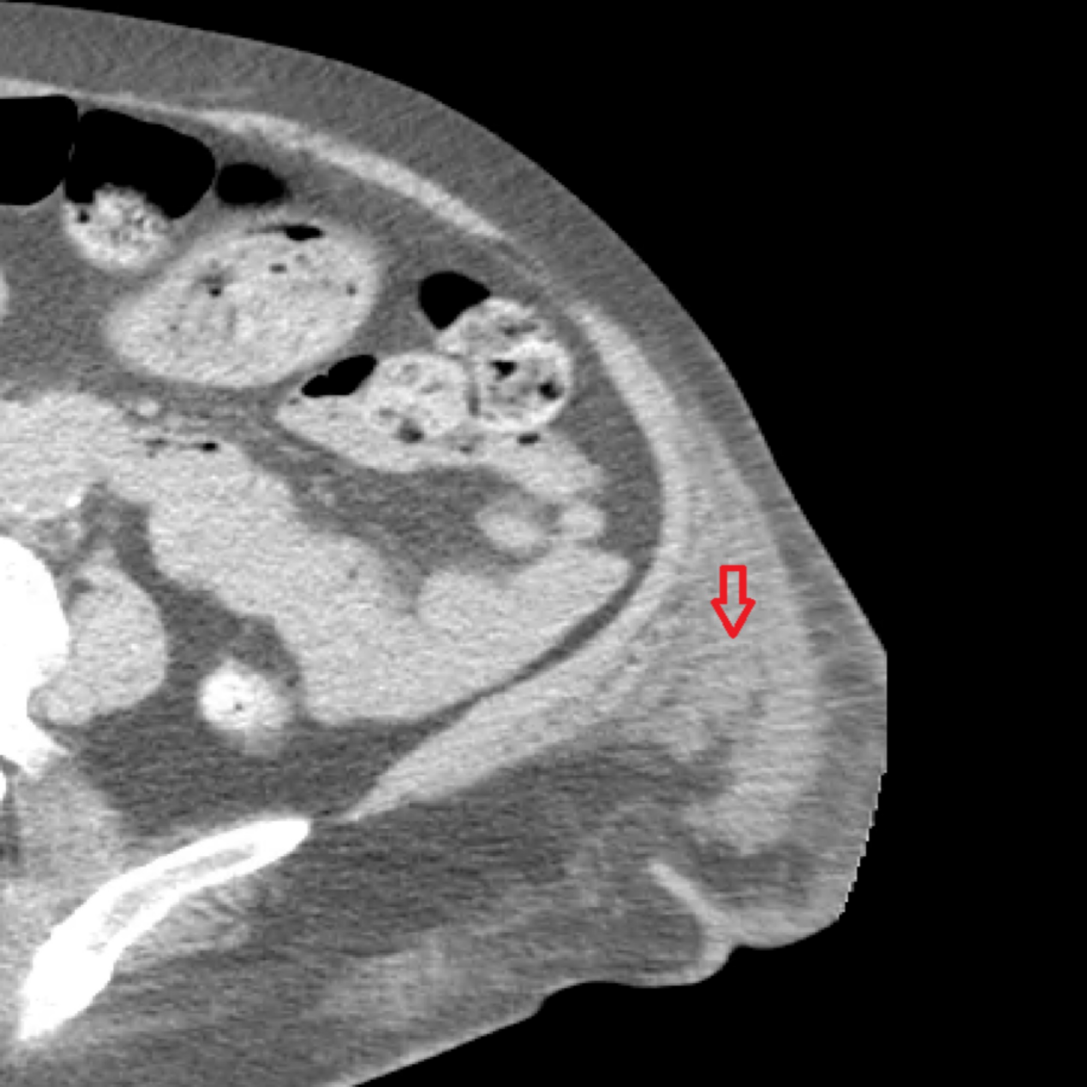
Arrow head indicating left thigh hematoma.

Initial laboratory workup showed low hemoglobin (5.1 mg/dL) and hematocrit (16.4%). White blood cell count and platelet count were normal (7000/uL and 288,000/uL respectively). Her coagulation profile showed increased partial thromboplastin time (81.8 s) but normal prothrombin time (11.5 s) and international normalized ratio (1). Activity testing for coagulation factors (IX, XI, XII) was within normal range except Factor VIII activity, which was <6%. CT with contrast revealed a 69 mm x 89 mm complex fluid collection in her left medial thigh musculature, suspicious for hematoma (Figure 1).

**Figure 2:**
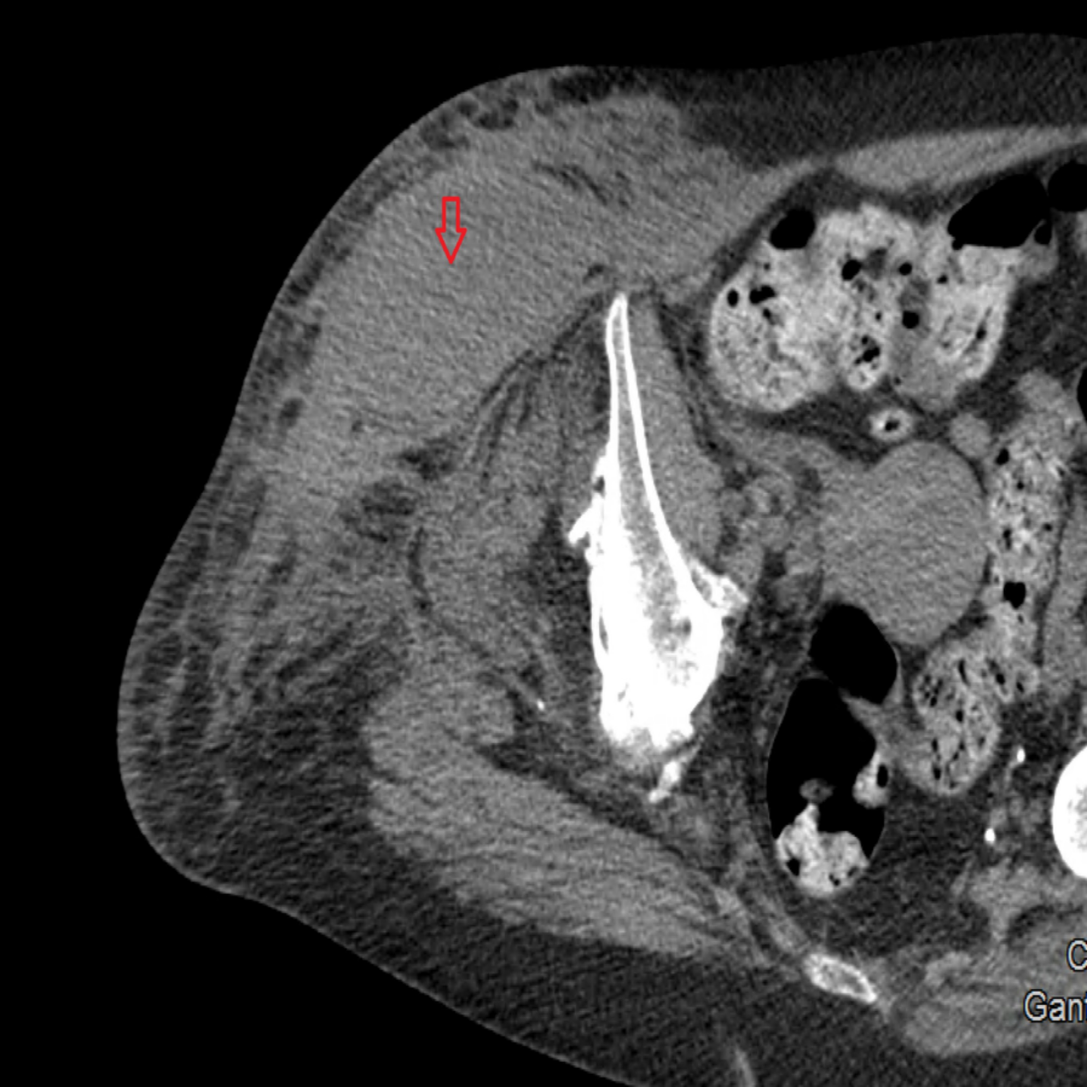
Arrow head indicating right thigh hematoma.

The patient was treated with packed red blood cells to maintain hemoglobin above 7 mg/dL, vitamin K (10 mg daily), fresh frozen plasma (two units daily), and Factor VIII concentrate (80 units/kg daily). Later, a mixing study was performed, indicating that the bleeding abnormality was due to Factor VIII inhibitor or lupus anticoagulant -- the latter of which was ruled out with a negative dilute Russell's viper venom time test. The treatment plan was modified to include the following: FEIBA, vitamin K (10 mg daily), and IV solumedrol (30 mg every 12 hours). Fresh frozen plasma was discontinued. Three days after starting FEIBA the patient started to complain of pain in her right groin. A stat CT of her right lower extremity showed a large hematoma in her right lateral thigh with a dimension of 7.9 cm x 10.1 cm axially and 19.3 cm craniocaudally (Figure 2).

She was subsequently transferred to the ICU for closer monitoring, during which her hemoglobin was 8.1 mg/dL. About half an hour after being transferred, she started to complain of chest pain. Electrocardiogram showed nonspecific T wave changes. Troponin was normal (8 pg/mL) and D-dimer was 17,000 ng/mL. During this event, the patient became hypotensive with a systolic blood pressure of 65 mmHg. She was also tachypnea with a respiratory rate of 44 breaths/minute. Her blood pressure was nonresponsive to IV fluid resuscitation and IV vasopressors. Thus, she was intubated for mechanical ventilation. Bedside echocardiogram showed a hyperdynamic left ventricular ejection fraction of approximately 70% +/- 5% with impaired diastolic dysfunction. Pulmonary embolism was the most suspected diagnosis at the time given the constellation of her symptoms and clinical signs. CT angiography of chest could not be performed due to the patient's unstable clinical condition. The patient was made comfort care by her family due to her poor clinical status. The patient passed away within 30 minutes of medical care withdrawal.

## Discussion

Hereditary hemophilia A is an inherited bleeding disorder in which there are reduced or absent levels of clotting Factor VIII, putting affected people at increased bleeding risk due to inadequate blood coagulation. AHA also leads to decreased Factor VIII but is caused by autoantibodies against the coagulation factor rather than a genetic deficiency [2]. The exact etiology behind the formation of autoantibodies against Factor VIII is unknown, but such autoantibodies have been linked to the presence of certain CTLA4 and HLA gene polymorphisms, leading to autoreactive CD4+ lymphocytes [3]. While the cause of AHA may be idiopathic, an association has been described with patients who are over the age of 50, have a penicillin allergy, having pregnancy, having autoimmune diseases, and having malignancy -- two of which were present in our patient [4]. 

Several well-known autoimmune conditions have been associated with AHA, including rheumatoid arthritis, systemic lupus erythematosus, and multiple sclerosis among several more. While these autoimmune diseases have been associated with AHA, the exact mechanism behind their association is unknown [4]. Furthermore, the autoimmune conditions in our patient (Addison’s disease and sarcoidosis) have not been described to be associated with AHA. Perhaps either or both of these conditions could have predisposed our patient with AHA, but this associated is currently unknown and not described in other literature. Thus, the etiology of AHA appears to be multifactorial, including idiopathic reasons, genetic predispositions, and environmental factors.

The clinical features of AHA include bleeding that occurs after a surgical procedure or that is unprovoked, leading to large hematomas, ecchymoses, or severe epistaxis, gastrointestinal bleeding, or hematuria. Spontaneous hemarthroses, which is commonly described in hereditary hemophilia A is unusual in those with AHA, but which occurred in our patient. The hemorrhagic symptoms can be severe, requiring emergency medical treatment [1]. A workup for an acquired Factor VIII inhibitor should be considered in patients who present with these symptoms with no known underlying reason. Diagnosis of AHA is confirmed with prolonged aPTT and normal PT studies, a mixing study in which there is persistent prolongation of aPPT. Von Willebrand disease and bleeding due to heparin should be ruled out as well [5]. 

While desmopressin may be used to control non-life-threatening bleeding, the superior efficacy of factor bypassing agents is recommended in most cases. The two mainstay bypassing agents recommended are FEIBA and recombinant Factor VIIa, both of which have been shown to overcome the antibody’s inhibiting effect on hemostasis. FEIBA contains unactivated Factors II, IX, and X; as well as activated Factor VII. The presence of activated Factor VII can directly activate Factor X. Because Factor X is more downstream in the coagulation pathway than Factor VIII, this process bypasses the need for Factor VIII and restores thrombin generation needed to form a fibrin clot [6]. FEIBA has been shown to effectively control bleeding in AHA at a rate of 93.3% [7]. While treatment has been effective, the current recommended FEIBA infusion rates used in practice (3.8 U/kg/min) are substantially higher than that recommended in the Summary of Product Characteristics (SmPC) for FEIBA (2.0 U/kg/min). The reason and consequence of this discordance are unknown [8]. 

While FEIBA may be an effective method in restoring clot formation in AHA undergoing a bleeding event, an unfortunate adverse effect of thromboembolism may occur and has been documented as in our patient. While thromboembolism has been a reported adverse effect in FEIBA therapy, it is quite rare. Per data that was collected in the European Acquired Haemophilia (EACH2) Registry, thrombotic events occurred 4.8% of the time in AHA patients who received FEIBA [9]. The most common thromboembolic events associated with FEIBA include deep vein thrombosis, pulmonary embolism, disseminated intravascular coagulation, and myocardial infarction. While the exact etiology for thrombosis occurring in these patients is unknown, factors such as duration of treatment, prolonged bed rest, and hepatic, cardiovascular, and metabolic disorders may increase a patient’s risk for a thrombotic adverse effect due to FEIBA therapy [10].

## Conclusions

Although thromboembolism in patients receiving FEIBA is rare, it is still a complication that can occur and is life-threatening to the patient and the benefits and risks should be carefully evaluated by clinicians before initiating treatment. Additionally, the discordance between actual and recommended infusion rates along with and potential life-threatening risk of thromboembolism leaves more to be desired in terms of understanding the exact safety profile of FEIBA. Thus further, large scale studies focusing on the safety of FEIBA should be investigated to minimize potential adverse effects in patients with AHA.
